# Experimental study of an evanescent-field biosensor based on 1D photonic bandgap structures

**DOI:** 10.3762/bjnano.10.97

**Published:** 2019-04-26

**Authors:** Jad Sabek, Francisco Javier Díaz-Fernández, Luis Torrijos-Morán, Zeneida Díaz-Betancor, Ángel Maquieira, María-José Bañuls, Elena Pinilla-Cienfuegos, Jaime García-Rupérez

**Affiliations:** 1 Nanophotonics Technology Center, Universitat Politècnica de València, Camino de Vera s/n, 46022 Valencia, Spain; 2 Departamento de Química, Instituto Interuniversitario de Investigación de Reconocimiento Molecular y Desarrollo Tecnológico IDM, Universitat Politècnica de València, Camino de Vera s/n, 46022 Valencia, Spain

**Keywords:** evanescent field, half-antibodies, light-assisted immobilization, photonic bandgap sensor, SNOM characterization

## Abstract

A photonic bandgap (PBG) biosensor has been developed for the label-free detection of proteins. As the sensing in this type of structures is governed by the interaction between the evanescent field going into the cladding and the target analytes, scanning near-field optical microscopy has been used to characterize the profile of that evanescent field. The study confirms the strong exponential decrease of the signal as it goes into the cladding. This means that biorecognition events must occur as close to the PBG structure surface as possible in order to obtain the maximum sensing response. Within this context, the PBG biosensor has been biofunctionalized with half-antibodies specific to bovine serum albumin (BSA) using a UV-induced immobilization procedure. The use of half-antibodies allows one to reduce the thickness of the biorecognition volume down to ca. 2.5 nm, thus leading to a higher interaction with the evanescent field, as well as a proper orientation of their binding sites towards the target sample. Then, the biofunctionalized PBG biosensor has been used to perform a direct and real-time detection of the target BSA antigen.

## Introduction

The development of integrated lab-on-a-chip (LOC) devices for the highly sensitive and label-free detection of target analytes is one of the fields arousing high interest over the recent years [1–2]. These devices aim at overcoming some of the drawbacks of the methodologies commonly used today, such as ELISA, western blot or PCR tests, which typically are time-consuming, require several sample preparation steps (including labeling), have a high cost per assay, and need bulky lab-centralized instrumentation.

Integrated photonics is one of the technologies with a high potential for the development of LOC devices, due to the various advantages it provides, e.g., high sensitivity, miniaturization, high multiplexing level, fast response, need for very low sample and reagent volumes and the compatibility to complementary metal-oxide semiconductor (CMOS) fabrication [3]. Chip-integrated photonic biosensors have been demonstrated for several applications such as medical diagnosis, environmental monitoring or security control [4]. Most typical configurations of integrated photonic biosensors are based on the use of resonant or interferometric configurations, as it is the case of ring resonators or disks and Mach–Zehnder interferometers or bimodal waveguides, respectively. By using this type of structures, outstanding results have been obtained for the specific detection of a wide range of analytes, reaching detect limits even below the nanogram-per-milliliter range for protein detection [5] and below the femtomolar range for oligonucleotides detection [6].

A particular type of photonic sensing devices are photonic bandgap (PBG) biosensors based on evanescent-wave detection [7]. PBG structures consist of a periodic dielectric configuration for which the propagation of a certain wavelength range is forbidden because of that periodicity, the so-called PBG. The position of that PBG will depend on the interaction of the evanescent field of the Bloch modes in the structure with the surrounding medium. Additionally, note that Bloch modes at the edge of the PBG region exhibit a reduction of the group velocity that increases the interaction with the target analytes and thus the sensitivity. In comparison with resonant and interferometric configurations, PBG structures provide a higher degree of freedom in the design of their structural parameters. This might lead to a significant increase of the sensitivity and of the interaction mechanism with the surrounding medium, while keeping very small footprints (below 100 µm^2^).

In order to provide specificity to these photonic transduction elements, their surface must be biofunctionalized to immobilize bioreceptors being specific to the analytes of interest. Therefore, biofunctionalization is a key step for providing a label-free biosensing device able to analyze a chemical or a biological sample without requiring any pre-treatment (as for example labeling). Since the operation of photonic biosensors based on evanescent waves relies on the interaction between the target analytes and that part of the optical mode going into the cladding, it is important to maximize that interaction. This is achieved for distances closer to the sensor surface, where the intensity of the evanescent field is higher. Therefore, to achieve an optimum biodetection performance, it is important to consider biofunctionalization procedures that allow one to obtain biorecognition layers being as thin as possible and with robust links to the surface. This can be achieved, for example, by using covalent biofunctionalization strategies [8].

In this paper, we report the experimental work carried out to characterize and enhance the interaction with the target analytes in evanescent-wave-based sensors, specifically for the case of PBG sensing structures, and thus to increase the sensitivity of the sensors. The evanescent field of this type of sensing structures has been thoroughly characterized using scanning near-field optical microscopy (SNOM) in order to determine how the interaction will vary with the distance to the sensor surface. This near-field characterization has demonstrated the importance of having biorecognition layers being as thin as possible in order to reach optimal sensitivities. Taking this requirement into account, the PBG structures have been biofunctionalized using a light-assisted biofunctionalization protocol allowing for the covalent immobilization of half-antibodies, so that the thickness of the biorecognition layer can be significantly reduced. This biofunctionalization process was already demonstrated in [9–10], where the light-assisted immobilization of the half-antibodies was monitored in real time and in-flow using PBG sensing structures; however, a low sensing response towards antigen detection was obtained when using that in-flow biofunctionalization implementation. Therefore, in this work we have used an off-flow spotting process for the immobilization of the half-antibodies, which yields a higher surface coverage and thus significantly higher sensing responses, as demonstrated in the reported bovine serum albumin (BSA) detection experiments.

## Results and Discussion

### PBG sensing structures

**Figure 1:**
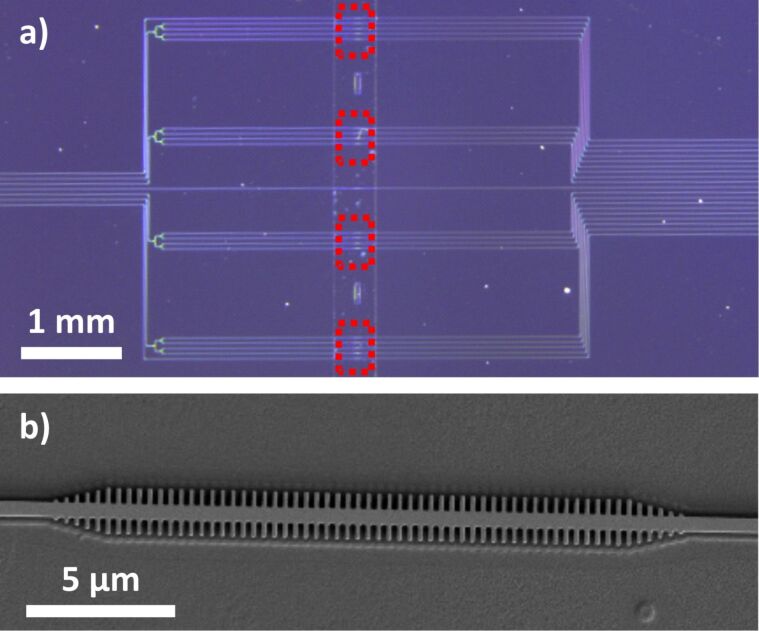
(a) Optical microscopy image of the fabricated photonic chip. Each of the PBG sensors groups are marked with a red dotted square. (b) Scanning electron microscopy image of one of the PBG sensing structures within the chip.

The PBG structures used in this work consist of a 1D silicon periodic configuration realized by the introduction of straight transversal elements in a conventional single-mode waveguide, as previously reported in other works [9–12]. The selected parameters for the silicon PBG structure are: height *h* = 220 nm, waveguide width *w* = 450 nm, period *a* = 380 nm, transversal elements length *w*_e_ = 1500 nm and transversal elements width *w*_i_ varying from 80 to 140 nm in order to tune the position of the PBG edge between 1500–1600 nm. The selected configuration of the PBG sensing structure was fabricated in a silicon-on-insulator (SOI) chip in our clean-room facilities (Figure 1). The created chip layout contains four groups of sensors, each one comprising four PBG structures where the width of the transversal elements has been swept between 80 and 140 nm (*w*_i_ = 80, 100, 120 and 140 nm for each of the PBG structures within each group) in order to ensure that the fabricated structures present their PBG edge within our measurement range as well as to be able to compare the sensing performance for slight variations of this parameter. The PBG structures are made of 60 transversal elements, including a linear taper of five elements at the accesses, leading to a total length of only ca. 22.8 µm and a footprint of only ca. 34.2 µm^2^. Each PBG sensors group is excited using the same access waveguide, which is divided using multimode interference (MMI) splitters.

### Evanescent field characterization

**Figure 2:**
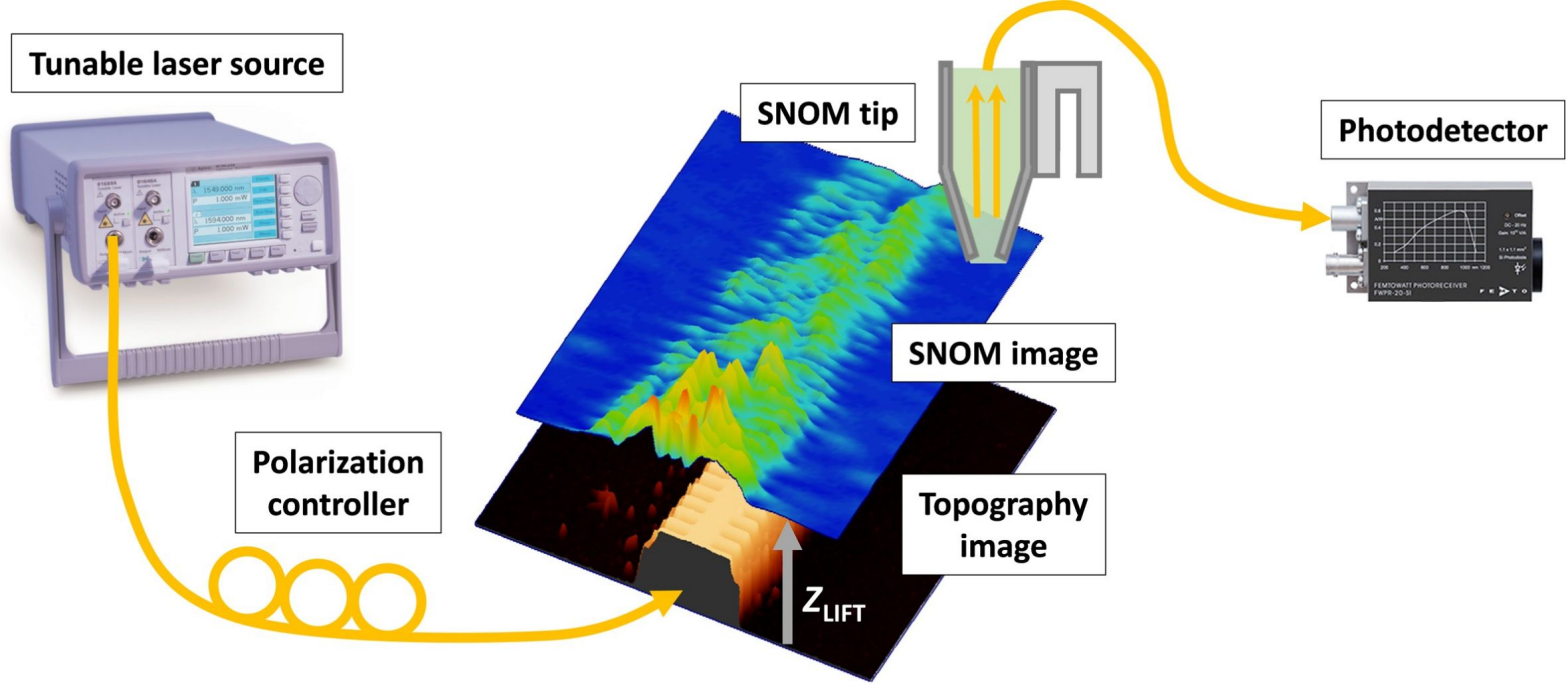
Schematic view of the SNOM characterization setup and measurement process.

Given the importance of the evanescent field profile on the sensing performance, the near-field behavior of the PBG sensing structures has been studied using SNOM. Figure 2 schematically depicts the tailored SNOM characterization setup used in this work, where light from a tunable laser was coupled to the photonic chip using a cleaved single-mode optical fiber and the near-field signal from the PBG structure was collected using a bent fiber tip pre-mounted on a tuning fork working in tapping mode.

**Figure 3:**
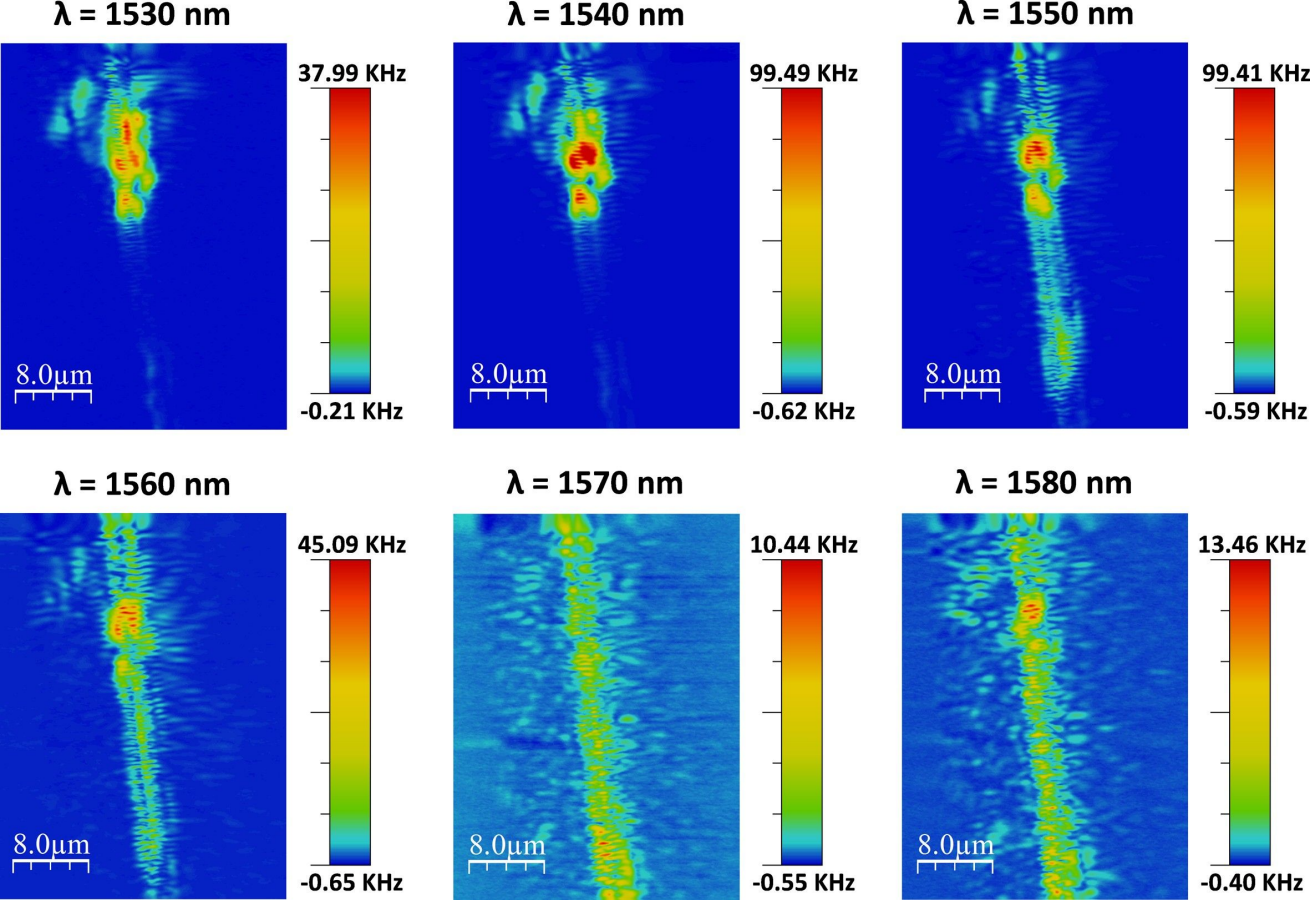
SNOM measurements obtained at different wavelengths (from 1530 to 1580 nm) for one of the PBG sensing structures having transversal elements of width *w*_i_ = 120 nm. The measurements are carried out at 20 nm from the surface of the PBG structure.

Figure 3 shows the SNOM signal measured for one of the PBG sensing structures having transversal elements of width *w*_i_ = 120 nm for different excitation wavelengths and for an upper cladding of air. Excitation wavelengths between 1530 nm and 1580 nm have been considered in the measurements. SNOM images obtained for wavelengths of 1530 and 1540 nm show a high back-scattering signal at the input of the PBG structure and no transmission was observed through the structures, thus confirming the presence of the PBG region. For wavelengths from 1550 to 1580 nm, transmission over the whole PBG structure can be observed, thus indicating that the Bloch modes of the structure are being excited and that we are out of the PBG region. Finite-difference time-domain (FDTD) simulations carried out for this configuration of the PBG structure predicted a PBG edge location at ca. 1530 nm, so a deviation of only about 20 nm was observed for the actual fabricated structure, i.e., ca. 1530 nm instead of ca. 1550 nm. This small deviation between the theoretical and the experimental response might be due to slight variations in the dimensions of the fabricated structure (of only few nanometers) or to the presence of SiO_2_ residues from opening the channel on the chip.

**Figure 4:**
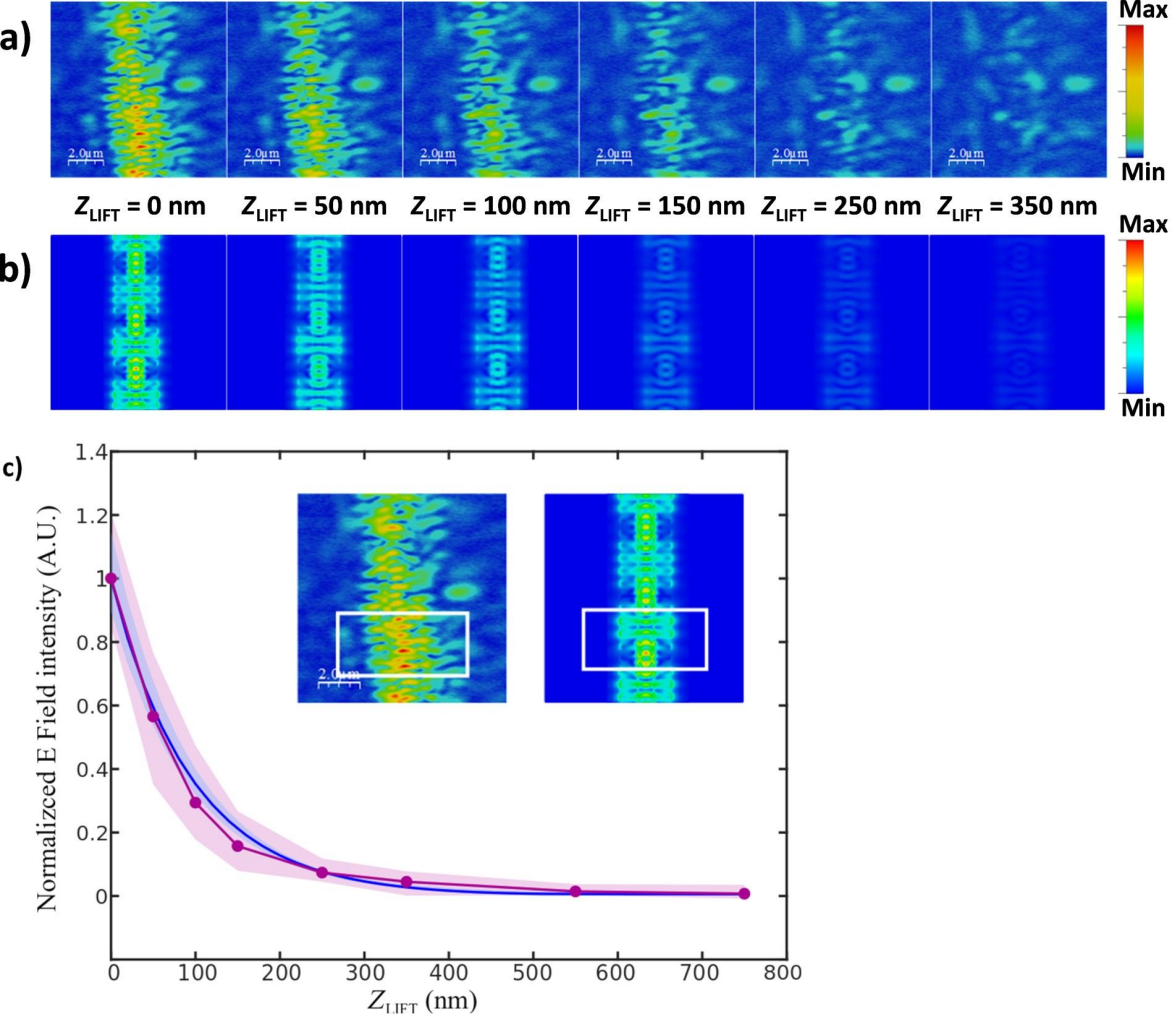
(a) SNOM-measured and (b) FDTD-simulated near field at different vertical distances (*Z*_LIFT_) from the surface of the PBG sensing structure. An excitation wavelength of 1570 nm is used for the SNOM measurements, while a wavelength of 1550 nm has been used in the FDTD simulations in order to consider the same scenario. (c) Normalized intensity of the evanescent field as a function of the distance from the sensor surface for the SNOM measurements (purple) and for the FDTD simulations (blue). The shaded areas represent the standard deviation of the raw SNOM measurements and the FDTD simulations along the area of the PBG structure highlighted in the insets.

Once the near-field behavior of the PBG sensing structure was characterized depending on the operation wavelength, i.e., inside or outside the PBG, the next step was measuring the decay length of the evanescent field in order to characterize how the sensing interaction will vary with the height. Figure 4a shows the SNOM measurements performed at different distances from the surface of the PBG structure for an excitation wavelength of 1570 nm. This excitation wavelength has been selected because it provides the best coupling to the PBG structure, as some back-scattering is observed at its access for all other excitation wavelengths (see Figure 3). SNOM images in Figure 4a show how the intensity of the evanescent field significantly decreases for heights above 100 nm. FDTD propagation simulations were then also carried out and the evanescent field intensity was calculated for the same heights used in the SNOM measurements, as shown in Figure 4b. Note that an excitation wavelength of 1550 nm was used for the FDTD evanescent field characterization in order to consider the same scenario than in the experimental SNOM characterization, i.e., a distance of 20 nm from the PBG edge. Both for the SNOM measurements shown in Figure 4a and for the FDTD simulations shown in Figure 4b, maximum values of the evanescent field intensity over different periods of the PBG structure were obtained. The results are presented in Figure 4c as a function of the distance from the surface. As it can be observed in the graph, a strong exponential decrease of the evanescent field intensity is observed, thus confirming the necessity of performing the biodetection as close to the surface as possible for the highest sensitivity. Note also the perfect agreement between the experimental SNOM characterization and the FDTD simulations.

**Figure 5:**
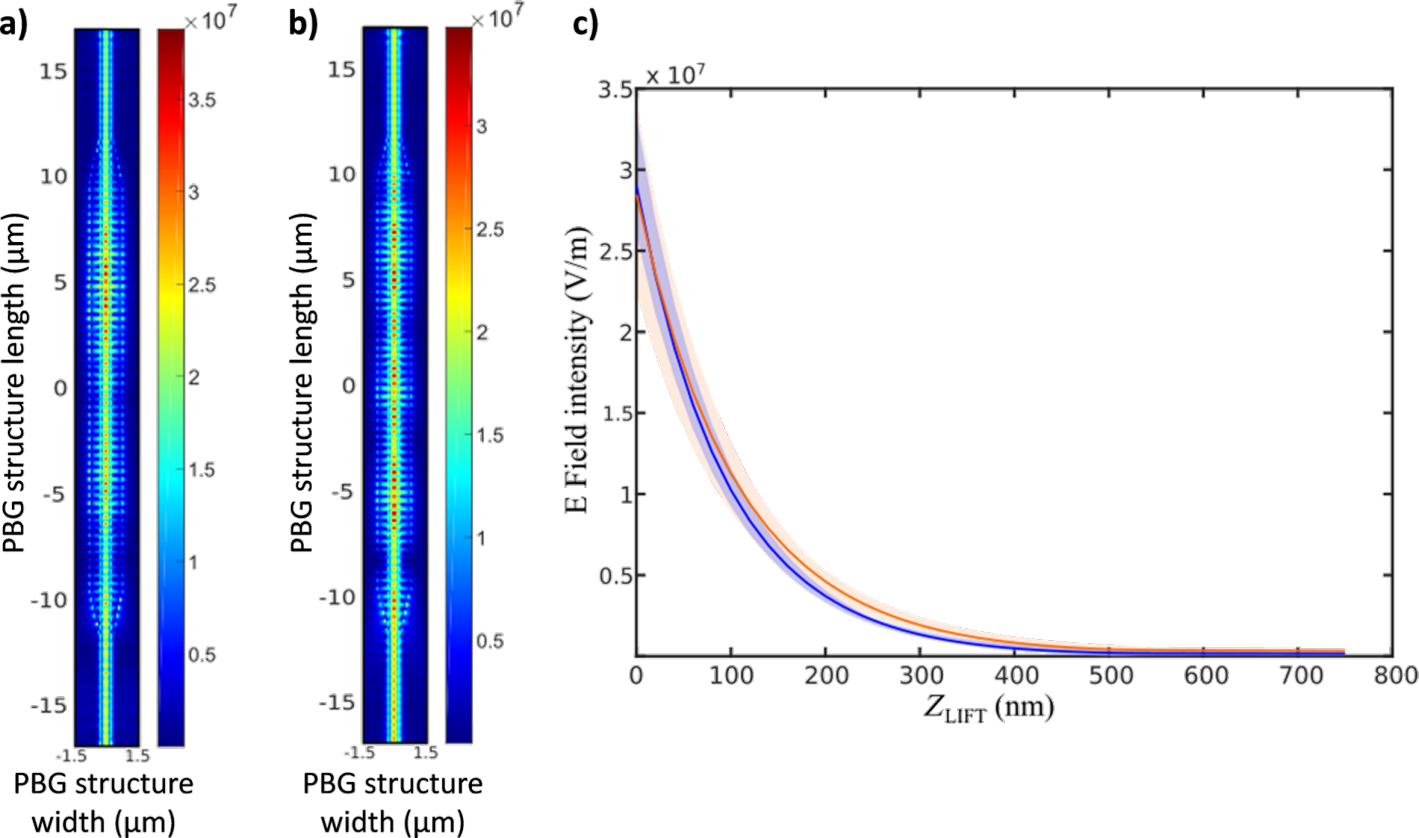
FDTD-simulated electric near-field intensity (V/m) through the PBG structure for (a) an upper cladding of air (λ = 1550 nm) and (b) an upper cladding of water (λ = 1600 nm). (c) Variation of the electric field intensity as a function of the distance to the sensor surface for the FDTD simulations when considering an air (blue) and a water upper cladding (red). As for Figure 4, the shaded areas represent the standard deviation of the intensity along the PBG structure.

Since our SNOM system does not allow us to perform near-field measurements of the photonic structures when having an upper cladding of water, and considering the good agreement between SNOM experiments and FDTD simulations previously shown, we have used FDTD simulations to determine the evanescent field profile in the scenario of water cladding. Figure 5 shows the electric-field profile simulated when considering upper claddings of air or water, as well as the variation of its intensity as a function of the distance to the sensor surface for these two scenarios. Note that, in order to consider an equivalent situation, both simulations have been made considering an excitation wavelength located at about 20 nm from the selected PBG edge (i.e., 1550 nm for an air upper cladding and 1600 nm for a water upper cladding). As it can be observed, the decay profile of the evanescent field in air and in water is almost the same.

### Biofunctionalization process

**Figure 6:**
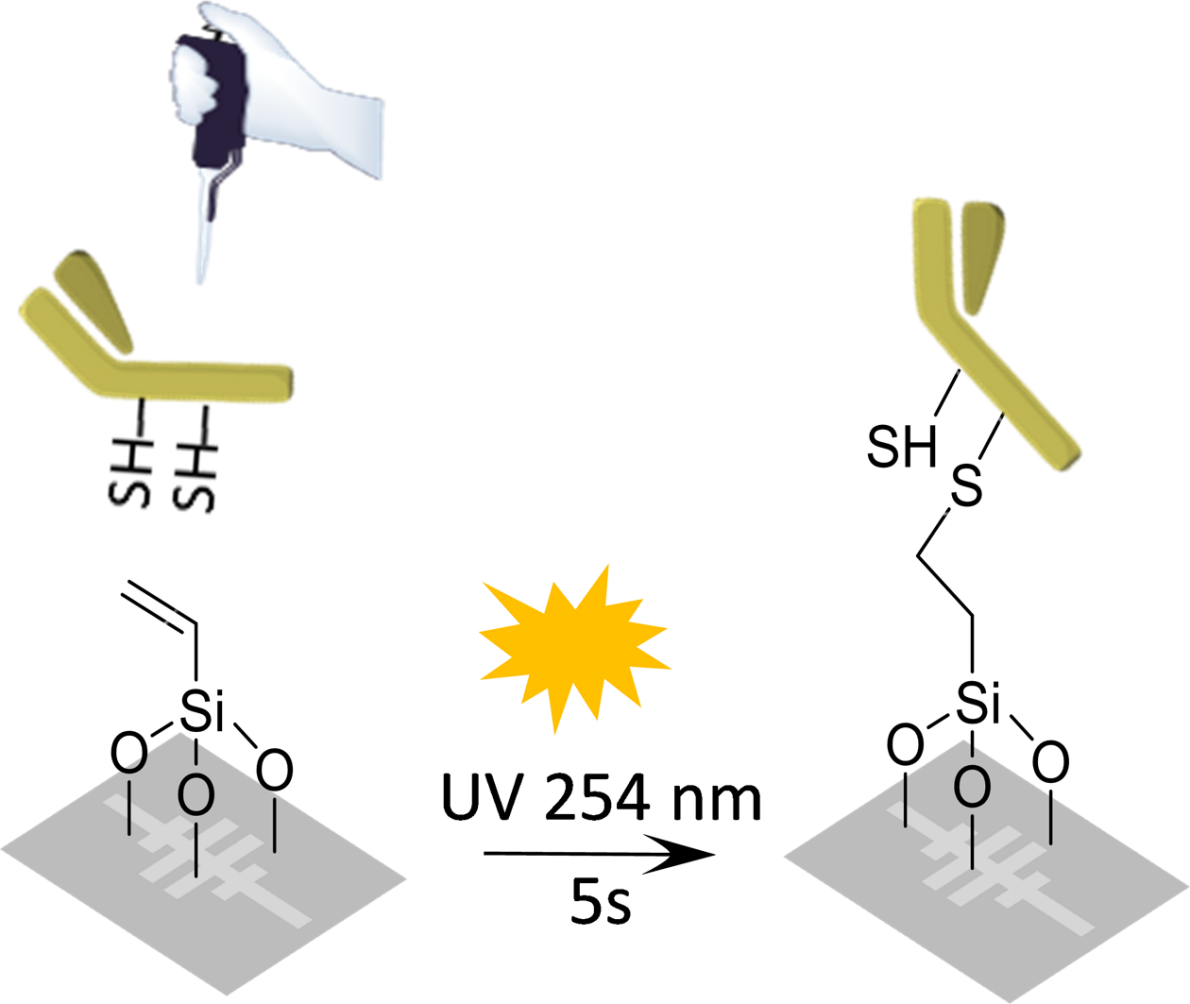
Schematic representation of the light-assisted immobilization of haBSA having free thiol moieties onto the vinyl-functionalized surface of the PBG sensing structures.

As determined from the evanescent field characterization performed by means of SNOM measurements and FDTD simulations, a very thin biorecognition layer is required for an optimum interaction of the biosensor with the target analytes. In order to achieve such a thin biorecognition layer, we have developed a light-assisted biofunctionalization approach that allows for the covalent immobilization of thiol-terminated bioreceptors onto a vinyl-terminated surface [13]. Half-antiBSA-antibodies (haBSA) have been used as bioreceptors in this work, which reduces even more the distance between the sensor surface and the recognition sites, and provides a proper orientation of these recognition sites. This biofunctionalization process, as well as the protocol for obtaining the half-antibodies, was previously described in [9–10], where the light-assisted immobilization of the half-antibodies was monitored in real time and in-flow using the PBG sensing structures. However, the sensing performance obtained for those experiments was relatively low, mainly determined by the low irradiation efficiency obtained when illuminating the photonic chip with UV light through the fluidic elements used to perform the experiments. So, in this case we have performed an off-flow immobilization of the haBSA, which were spotted over the PBG sensing structures on the silanized SOI chip (Figure 6), in order to obtain a better surface coverage leading to a higher sensitivity. By considering the thickness of the initial organosilane layer (ca. 4.5 nm, obtained from ellipsometry measurements) and by estimating the thickness of the half-antibodies deposited on the surface (ca. 2.5 nm), we determine that a total thickness of the whole biorecognition layer of only ca. 7 nm is achieved. When determining the immobilization density by fluorescence microarray measurements, densities corresponding to a close-packed monolayer of the half-antibodies were obtained with standard deviation of 8% indicating a good reproducibility of the immobilization method [13].

### Biosensing experiment

**Figure 7:**
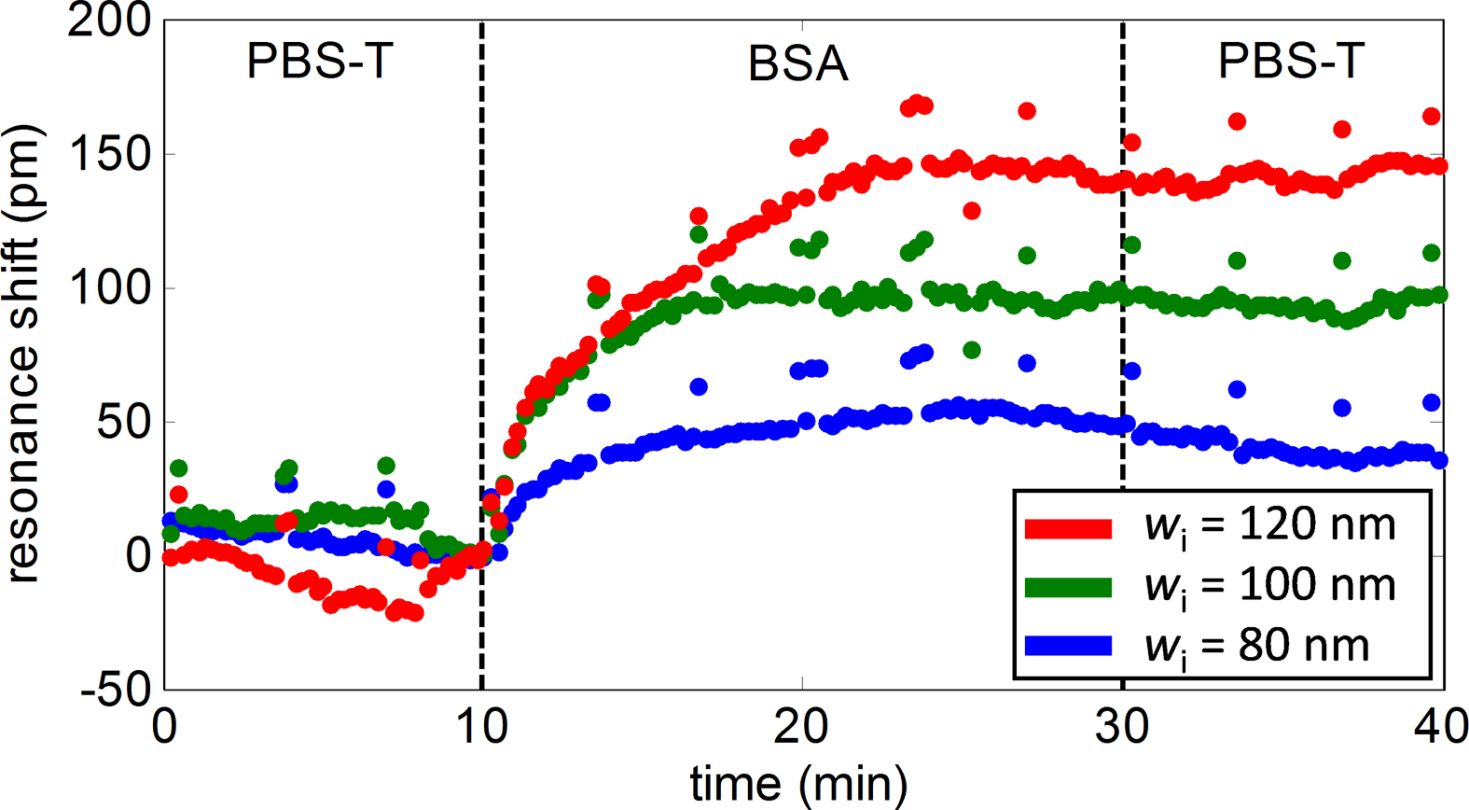
Time evolution of the PBG sensing response during the BSA detection experiment for one representative group of PBG sensing structures within the chip. Note that the PBG edge of the structures having *w*_i_ = 140 nm fell out of our characterization range, so it was not possible to measure the corresponding sensing response.

Figure 7 shows the results obtained for one of the groups of PBG sensing structures in the biosensing experiments carried out. After an initial flow of PBS-T buffer (phosphate buffered saline + 0.01% Tween 20) over a period of 10 min to establish the baseline, BSA 1 µg/mL in PBS-T was flowed over the biofunctionalized PBG sensing structures for 20 min for the real-time detection of the target antigens. Then, PBS-T buffer was flowed again for 10 min to remove excess BSA. As it can be observed in Figure 7, the target BSA was successfully detected by the PBG sensing structures, obtaining shifts of 50, 95 and 140 pm for structures having *w*_i_ values of 80, 100 and 120 nm, respectively. These sensing results mean a significant improvement of our previous results obtained using an in-flow immobilization of haBSA, where shifts below 20 pm were measured for a PBG structure with *w*_i_ = 120 nm [9]. This confirms the higher surface coverage obtained when using an off-flow spotting immobilization method. From the experimental results, we can also observe the great influence of the parameters of the PBG structure on its sensitivity, obtaining an increase of the shift of 45 pm every time the width parameter w_i_ is increased by 20 nm.

## Conclusion

We have developed a photonic biosensor based on PBG sensing structures for the specific detection of proteins. First, we have performed a study of the evanescent field profile in these sensing structures, since its interaction with the target analytes will determine the sensing performance of the structure. A strong exponential decay has been observed in the SNOM characterization carried out, thus highlighting the importance of having biorecognition layers as thin as possible for a high-sensitivity detection. Additionally, an almost perfect agreement has been observed between SNOM experimental measurements and FDTD simulations, thus making this simulation method appropriate for characterizing evanescent field photonic sensors with a high accuracy.

In order to fulfill the requirement of having a very thin biorecognition layer, we implemented a biofunctionalization protocol in which we combined a light-assisted immobilization protocol based on the TEC reaction with the use of half-antibodies. This biofunctionalization protocol allowed us to obtain a biorecognition layer of only ca. 7 nm allowing for a maximum interaction between the evanescent field and the target analytes. By means of BSA detection experiments, we have confirmed that a high sensitivity is obtained. We have also observed that this sensitivity might also be increased by properly tuning the structural dimensions of the PBG sensing structures, as simply an increase of the transversal elements width *w*_i_ yielded a sensitivity increase by a factor about three.

## Experimental

For the creation of the photonic chips, a standard nanofabrication process optimized in our clean-room facilities has been used [11]. E-beam lithography with 100 keV acceleration voltage was used to expose the created chip layout onto a 100 nm thick hydrogen silsesquioxane (HSQ) resist layer. Then, the layout was transferred to the top 220 nm thick silicon layer of the SOI chip by means of inductively coupled plasma etching. 70 nm deep shallow etch 1D grating couplers were created for accessing the photonic chip. Finally, the chip is covered with a 400 nm thick SiO_2_ upper cladding, and a 400 µm wide channel is opened on it using UV lithography in order to have access to the PBG sensing structures.

The SNOM measurements have been performed by using a customized MultiView 4000 system (Nanonics Imaging Ltd.) working in collection mode, which has been used in previous works to characterize SOI structures as nanoantennas [14–15]. In this system, a cleaved single-mode optical fiber was used to couple light from a tunable laser (Keysight 81980A) to the photonic structures on the chip via their input grating couplers. A bent fiber tip with a 500 nm aperture, Cr/Au coated, pre-mounted on a tuning fork working in tapping mode at 36.19 kHz and placed perpendicular to the sample was used to scan the photonic structures and measure the near-field signal using a FWPR-S femtowatt photoreceiver. A step size of 40 nm in the *xy*-plane was used when acquiring the SNOM image (i.e., ca. 10 points per period of the PBG structure). The whole system (photonic chip, input fiber and SNOM tip) can be previsualized with an optical microscope used to obtain a proper alignment of the input fiber as well as an accurate positioning of the SNOM probe.

Finite-Difference time-domain (FDTD) simulations have been carried out by using CST Microwave Studio simulation software.

The biofunctionalization of the photonic sensors using the light-assisted immobilization process developed by our group [9–10,13], started with a cleaning of the SOI photonic chip using piranha solution (H_2_SO_4_/H_2_O_2_ = 1:3) for 20 min and a subsequent activation of its surface with O_2_ plasma for 10 min. Then, the SOI chip was silanized by immersing it in 1% TEVS in Milli-Q water (pH adjusted to 8) for 1 h and finally curing it at 110 °C for an additional 1 h. The reduction process used to obtain the half-antibodies consisted on the incubation (90 min at 37 °C) of the BSA antibodies in acetate buffer (0.15 M sodium acetate, 0.01 M EDTA, 0.1 M sodium chloride, pH 4.5) at 4 mg/mL concentration in the presence of 25 mM tris(2-carboxyethyl)phosphine (TCEP). The reduced haBSA were purified using a 50 kDa centrifugal filter unit. For the immobilization of the half-antibodies, haBSA were spotted over the PBG sensing structures on the silanized SOI chip (4 μL of haBSA at 20 µg/mL in PBS 0.1× buffer) and then incubated for 30 min at 37 °C before the chip was irradiated with UV light (λ = 254 nm) for 5 s.

For the characterization of the photonic sensors, the SOI chip was assembled with a polydimethylsiloxane (PDMS) microfluidic flow cell and then placed in an opto-fluidic experimental setup allowing for the simultaneous interrogation of all photonic sensing structures within the chip [10–11]. In order to characterize the photonic chip, it was excited via their access grating couplers using light from a continuous-sweep tunable laser (Keysight 81980A) and the output light corresponding to each photonic structure within the chip was measured using an infrared camera (Xenics Xeva-1.7-320). These two elements were synchronized via a trigger signal in order to obtain the spectra of all the photonic structures simultaneously. A syringe pump working in withdraw mode was used to flow the target solutions over the sensing chip at a rate of 10 μL/min.
